# Rice and cold stress: methods for its evaluation and summary of cold tolerance-related quantitative trait loci

**DOI:** 10.1186/s12284-014-0024-3

**Published:** 2014-10-02

**Authors:** Qi Zhang, Qiuhong Chen, Shaoling Wang, Yahui Hong, Zhilong Wang

**Affiliations:** 1 Hunan Provincial Key Laboratory of Crop Germplasm Innovation and Utilization, College of Biological Science and Technology, College of Agronomy, Hunan Agricultural University, Changsha 410128, Hunan, China; 2 Department of Anatomy and Neurobiology, Xiangya School of Medicine, Central South University, Changsha 410013, Hunan, China

**Keywords:** Cold tolerance, Physiological metabolites, Evaluation criteria, QTL, Oryza sativa

## Abstract

Cold stress adversely affects rice (*Oryza sativa* L.) growth and productivity, and has so far determined its geographical distribution. Dissecting cold stress-mediated physiological changes and understanding their genetic causes will facilitate the breeding of rice for cold tolerance. Here, we review recent progress in research on cold stress-mediated physiological traits and metabolites, and indicate their roles in the cold-response network and cold-tolerance evaluation. We also discuss criteria for evaluating cold tolerance and evaluate the scope and shortcomings of each application. Moreover, we summarize research on quantitative trait loci (QTL) related to cold stress at the germination, seedling, and reproductive stages that should provide useful information to accelerate progress in breeding cold-tolerant rice.

## 1 Introduction

Crops are exposed to varied environmental conditions during their life cycle. Cold stress, which can be classified as chilling (0–15°C) and freezing (<0°C) stress, is a major environmental factor limiting the growth, productivity, and geographical distribution of crops (Zhu et al. [2007]). Rice (*Oryza sativa* L.), one of the most world’s most important staple crops, feeds more than 2.7 billion people worldwide and is extensively grown by more than half of the world’s farmers (Fairhurst and Dobermann [2002]; Shelton et al. [2002]). Due to its origin in tropical and subtropical regions, rice is more sensitive to cold stress than other cereal crops such as wheat (*Triticum aestivum* L.) and barley (*Hordeum vulgare* L.). Therefore, in temperate areas, the production of rice is severely limited by cold stress (Xie et al. [2012]). Low temperatures that occur at critical reproductive stages can adversely affect grain quality or cause yield reductions in high-latitude or high-altitude regions of China, Japan, Korea, and other parts of the world (Jena et al. [2012]).

Over the past 20 years, extensive efforts have been made to improve cold tolerance in rice, which is a very complex trait (Maruyama et al. [2014]). Cold stress affects chlorophyll content and fluorescence, and thus interferes with photosynthesis in rice (Kanneganti and Gupta [2008]; Kim et al. [2009]). Moreover, increased contents of reactive oxygen species (ROS) and malondialdehyde (MDA) that accumulate during cold stress in rice can impair metabolism *via* cellular oxidative damage (Xie et al. [2009]; Nakashima et al. [2007]). On the other hand, rice also possesses strategies to cope with or adapt to cold stress. For example, cold-treated rice plants accumulate proline, an amino acid that stabilizes protein synthesis, and thereby maintains the optimal function of rice cells (Kandpal and Rao [1985]). Under cold stress, contents of antioxidant species also increase to scavenge ROS and protect rice plants against oxidative damage (Sato et al. [2011]). Such physiological changes that occur upon cold treatment of rice, whether mediators or symptoms of cold damage, can also be used as indicators to evaluate the cold tolerance of rice.

Due to diverse growing locations and climatic factors, rice cultivars face cold stress at specific growth stages (Saito et al. [2001]). Researchers have established many growth-stage specific criteria to evaluate and select cold-tolerant rice. Evaluation of rice cultivars typically takes place during seedling and reproductive stages that are critical to production of rice. However, in high-latitude or high-altitude regions, low temperatures during long, cold springs can severely inhibit germination and constrain early seedling growth. So evaluation of cold tolerance at the germination stage is especially significant for these regions.

As the development of molecular markers and linkage maps progresses, marker-assisted selection becomes an effective way to breed cold-tolerant cultivars. Many cold-tolerance related QTL have been identified in the past 20 years. The QTL *Ctb1*, *qCTB2a*, *qPSST-3*, *qLTB3* are related to cold tolerance at the reproductive stage; *qCTP11* is related to cold tolerance at the germination stage; and *qCtss11* and *qCTS4a* are related to cold tolerance at the seedling stage. Because breeding for abiotic-stress resistance is urgently needed, progress in the identification of cold tolerance-related QTL has been a significant development for facilitating molecular marker-assisted selection (MAS).

In this review, we focus on clarifying the role of various metabolites during the response to cold stress in rice, and summarize the diverse criteria that are useful for evaluating the cold tolerance of rice at different growth stages. In addition, we discuss QTL and markers related to cold tolerance that can be used to facilitate marker-assisted breeding through recurrent selection in rice.

## 2 Review

### 2.1 Changes in physiological parameters of rice under cold stress

Low temperature not only inflicts obvious physical damage to rice plants, including low germination rate, stunted seedling growth, high death rate, and low spikelet fertility, but also initiates physiological fluctuations, such as increased electrolyte leakage (EL), changes in chlorophyll fluorescence, and increases in amounts of ROS, MDA, sucrose, lipid peroxidation, proline, and other metabolites. Analysis of metabolites in cold-stressed *Arabidopsis* by gas chromatography–mass spectrometry (GC-MS) has detected a total of 434 low-molecular-weight carbohydrates, amines, organic acids, and other polar molecules. Of these metabolites, levels of 325 (75%) had increased, and levels of 114 (35%) metabolites had increased at least 5-fold upon cold stress (Cook et al. [2004]). These metabolites could be organized into six categories related to 1) photosynthesis, 2) electrolyte leakage, 3) ROS and MDA, 4) stress-related soluble sugars, 5) cold-response related amino acids, and 6) antioxidants (Table 1). Thus, changes in the concentrations of these metabolites can be useful indicators for analysis of cold-stress responses in rice.

**Table 1:** Changes in metabolic properties of rice plants during cold stress

**Group and property**	**Effect on tolerance**	**Relevant gene**	**Reference**
**Photosynthesis**			
Chlorophyll content	Positive	*OsiSAP8*, *TERF2*	(Kanneganti and Gupta [2008]; Tian et al. [2011])
Fv/Fm	Positive	*CBF1/DREB1b*, *Asr1*, *OsCDPK7*	(Kim et al. [2009]; Lee et al. [2004]; Saijo et al. [2001])
**Electrolyte leakage**	Negative	*CBF1/DREB1b*, *OsLti6*, *ZFP245*, *TERF2*, *OVP1*, *OsNAC5*	(Huang et al. [2009]; Tian et al. [2011]; Song et al. [2011]; Zhang et al. [2011a]; Lee et al. [2004]; Morsy et al. [2005])
**ROS and MDA**			
Hydrogen peroxide	Negative	*OsAPXa*, *OsMKK6*, *OsMPK3*, *OsNAC6*, *OsTRX23*, *SodCc1*, *OsPOX1*	(Xie et al. [2012]; Xie et al. [2009]; Sato et al. [2011]; Lee et al. [2009]; Nakashima et al. [2007])
Superoxide radicals	Negative	*OsAPXa*	(Sato et al. [2011])
Hydroxyl radicals	Negative	*OsAPXa*	(Sato et al. [2011])
Malondialdehyde	Negative	*OsAPXa*, *OsMKK6*, *OsMPK3*, *OsNAC6*, *ZmCBF3*	(Sato et al. [2011]; Xie et al. [2012]; Xu et al. [2011]; Nakashima et al. [2007])
**Soluble sugars**			
Sucrose	Positive	*OSINV4*, *OsDREB1A*, *TERF2*	(Tian et al. [2011]; Oliver et al. [2005]; Ito et al. [2006])
Hexose	Positive	*OSINV4*	(Oliver et al. [2005])
Raffinose	Positive	*OsDREB1A*, *TERF2*	(Tian et al. [2011]; Ito et al. [2006])
Glucose	Positive	*OsDREB1A*, *TERF2*	(Tian et al. [2011]; Ito et al. [2006])
Fructose	Positive	*OsDREB1A*, *TERF2*	(Tian et al. [2011]; Ito et al. [2006])
Trehalose	Positive	*OsNAC5*, *OsPP1*, *OsPP2*, *OsTPP*	(Song et al. [2011])
**Cold-related amino acids**			
Proline	Positive	*OsCOIN*, *OsDREB1A*, *OsMYB2*, *OVP1*, *OsNAC5*, *MYB4*, *OsPRP3*, *ZFP245*, *OsMYB3R-2*	(Huang et al. [2009]; Tian et al. [2011]; Song et al. [2011]; Zhang et al. [2011a]; Liu et al. [2007]; Yang et al. [2012]; Vannini et al. [2004]; Gothandam et al. [2010]; Ma et al. [2009])
**Antioxidants**			
Ascorbic acid	Positive	Unknown	(Kim and Tai [2011])
Glutathione	Positive	*OsTRX23*	(Kim and Tai [2011]; Xie et al. [2009])

#### 2.1.1 Chlorophyll content and fluorescence indicate effects of cold stress on photosynthesis

Chlorophyll content and fluorescence, measured as the ratio of variable fluorescence to maximum fluorescence (F_v_/F_m_), are two photosynthetic properties whose changes are relevant to cold responses in plants. Chlorophyll content can signify nutrient stress in general, and nitrogen or sulfur stress in particular (Haboudane et al. [2002]). Cold stress can inhibit chlorophyll synthesis and chloroplast formation in rice leaves. Thus, reduced chlorophyll content can indicate the effect of low temperature on rice plants (Sharma et al. [2005]).

F_v_/F_m_ is a measure of chlorophyll fluorescence that is commonly used to determine the maximum quantum efficiency of Photosystem II (PSII), which indicates whether cold stress has compromised PSII in its dark-adapted state (McFarlane et al. [1980]). During cold stress, F_v_/F_m_ values decrease slightly in plants that tolerate cold, but decrease significantly in plants that are sensitive to cold (Bonnecarrère et al. [2011]; Zahedi and Alahrnadi [2007]). This parameter is thus useful for assaying the cold tolerance and sensitivity of plants that differ for this trait due either to genetics or acclimation.

The expression of several genes known to be involved in stress signal transduction can influence chlorophyll content or fluorescence. For example, chlorophyll content is much higher compared to that of control plants in both rice and tobacco transgenic lines overexpressing *OsiSAP8*, a member of the *SAP* gene family in rice. *OsiSAP8* encodes a cytoplasmic zinc finger protein that acts early in the signal transduction of responses to various stresses including cold (Kanneganti and Gupta [2008]).

**Figure 1:**
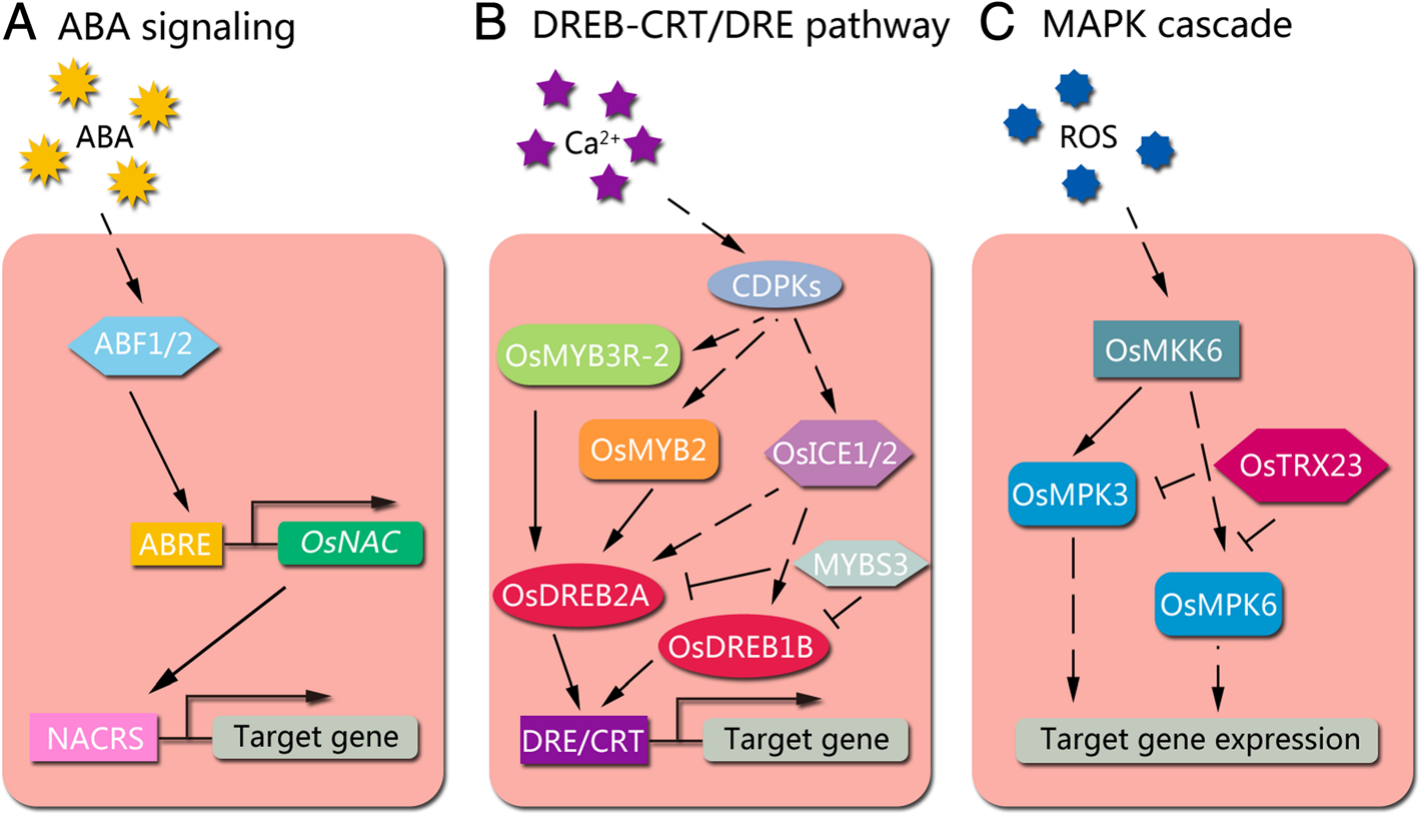
**Cold sensing and responsive pathway in rice. (A)** ABA signaling is initiated by ABA accumulation, and is transduced by ABF and the ABRE-containing *OsNAC* gene, which regulates the expression of a NACRS-containing target gene to increase cold tolerance. **(B)** The DREB-CRT/DRE pathway is initiated by a Ca^2+^-influx signal that is transduced by calcium-dependent protein kinases (CDPKs), OsMYB3R-2, OsMYB2, and OsICE1/2. The expression of *OsDREB1B* and *OsDREB2A* are down-regulated by MYBS3. **(C)** The MAPK cascade (OsMKK6-OsMPK3 and OsMPK6) is activated by ROS and is negatively regulated by OsTRX23. Solid arrows indicate direct activation; broken arrows indicate indirect activation; lines ending with a bar indicate negative regulation.

Additionally, ectopic expression of another cold-responsive gene, *OsAsr1*, which functions in abscisic acid (ABA)-response and fruit ripening, results in twofold higher values for F_v_/F_m_ in transgenic rice seedlings, a finding consistent with enhanced cold tolerance (Kim et al. [2009]). The phytohormone ABA is involved in various aspects of plant growth and development, and one of its major roles is to mediate adaptive responses to various environmental stresses (Hossain et al. [2010b]). In cold-stressed rice, ABA accumulates and initiates the ABA signaling cascade, which affects the expression of ABA-responsive genes *via cis*-acting ABA-response elements (ABRE) and the ABRE-binding bZIP transcription factor (ABF) (Hossain et al. [2010a]). The *OsNAC* gene transduces the ABA signal through an ABRE in its promoter and regulates the expression of NACRS-containing genes to control cold tolerance in rice (Figure 1A) (Nakashima et al. [2012]; Nakashima et al. [2007]; Song et al. [2011]).

Yamburenko reported that exogenous ABA reduced chlorophyll content and differentially affected the expression of chloroplast genes, suggesting a role of ABA in the regulation of proteins with functions in photosynthesis in barley leaves (Yamburenko et al. [2013]). However, in rice, the role of ABA in regulation of photosynthesis-related genes has not yet been fully explained. Therefore, progress in this field will help to bridge the gaps in our knowledge of the crosstalk between cold stress, ABA signaling, and photosynthesis.

#### 2.1.2 Changes in membrane fluidity initiate cellular cold responsive

Changes in ambient temperature can affect cell membranes quickly, although this is a reversible process (Murata and Los [1997]; Los and Murata [2004]; Sangwan et al. [2002]). Changes in membrane fluidity are measured by the membrane polarization index, *p*, an inverse indicator of membrane fluidity (Sangwan et al. [2002]).

Rice cells can sense cold stress based on changes in membrane rigidity, the physical state of membrane proteins, and osmotic pressure (Los and Murata [2004]). Low temperatures initiate increased membrane rigidity and can lead to increased EL, which can indicate the activity of cold tolerance-relevant genes, including *TERF2*, *OVP1*, and *OsNAC5* (Yun et al. [2010]; Tian et al. [2011]; Zhang et al. [2011a]; Song et al. [2011]). Functional characterization of the cold-activated *Stress-activated MAPK* (*SAMK*) reveals that a MAPK signaling cascade is triggered by increased membrane rigidity and altered ion conductance within cells and tissues (Sangwan et al. [2002]). Also, the influx of Ca^2+^ into the cytoplasm, an early event in cold stress, may be mediated by Ca^2+^ channels that are activated by membrane rigidification, ligands, or mechanical stimuli (Chinnusamy et al. [2006]). This cold-sensing signal can be interpreted and amplified by a calcium signaling cascade that subsequently activates the DREB-CRT/DRE (dehydration-responsive element-binding proteins-C-repeat/dehydration-responsive elements) pathway in rice (Zhang et al. [2013]), which is an important cascade for cold sensing and response in rice (Figure 1B) (Zhu et al. [2007]; Chinnusamy et al. [2007]).

#### 2.1.3 ROS and MDA mediate cold damage and cold sensing in rice

ROS are chemically reactive molecules that contain oxygen, including superoxide (O_2_^−^), hydrogen peroxide (H_2_O_2_), or the hydroxyl radical (HO^−^) that are produced at low levels as normal byproducts of plant cellular metabolism, mainly in organelles such as chloroplasts, mitochondria, and peroxisomes. However, both biotic and abiotic stresses can lead to excessive production of ROS that can then react rapidly with proteins, DNA, and lipids to cause cellular oxidative damage (Apel and Hirt [2004]; Skopelitis et al. [2006]; Mittal et al. [2012]).

In chloroplasts, ROS may cause over-reduction of the electron transport chain, limit CO_2_ fixation, and interfere with the photosynthetic process. ROS can also cause damage during stress through their effects upon the electron transport chain in mitochondria (Suzuki and Mittler [2006]). ROS degrade polyunsaturated lipids to form MDA, a reactive aldehyde that initiates toxic stress in cells and subsequently causes cellular dysfunction and tissue damage (Pamplona [2011]). In a study evaluating QTL associated with cold tolerance, elevated MDA content (0.00–0.57 nmol g^−1^ FW) was found in 92.16% (47/51) of rice cultivars (Kim and Tai [2011]). Other studies have revealed that the accumulation of ROS in cells of cold-treated rice triggers expression of cold-responsive genes and regulation of the cold-responsive signaling network *via* the OsMKK6-OsMPK3 (MAPKK-MAPK) pathway (Figure 1C) (Xie et al. [2009]).

#### 2.1.4 Soluble sugars, proline, and antioxidants protect rice from further damage due to cold stress

Soluble sugars that accumulate in plants under stress include sucrose, hexose, raffinose, glucose, fructose, and trehalose. These sugars act as compatible solutes in freezing stress, serving as osmoprotectants against freezing-dehydration damage (Nagao et al. [2005]; Shao et al. [2007]; Yuanyuan et al. [2010]). Because sucrose, trehalose, raffinose, and stachyose contents can increase under low temperature, these metabolites can be used as indicators to evaluate the potential cold tolerance of rice varieties (Morsy et al. [2007]).

Moreover, proline accumulation is also enhanced by cold stress. In addition to acting as a reservoir of carbon and nitrogen, proline also protects cellular enzymes from denaturation (Shah and Dubey [1997]). Proline stabilizes the polyribosome and thereby maintains the operation of protein synthesis (Kandpal and Rao [1985]). Proline is also involved in removal of stress-related excess H^+^ and maintains oxidative respiration at optimal cytosolic pH (Venekamp [1989]). Moreover, proline increases protein water-binding ability through its hydrophobic interactions with the surface residues of proteins (Schobert and Tschesche [1978]). Increased proline content has been widely observed in rice varieties under low temperatures. Finally, the significant correlations between proline contents and cold tolerance have help to confirm the function of proline during the cold response in rice (Kim and Tai [2011]).

In another metabolic adaptation, antioxidant species scavenge ROS to protect rice plants against oxidative damage induced by cold stress. These antioxidant species include ascorbic acid (AsA) and glutathione (GSH) (Kim and Tai [2011]; Xie et al. [2009]). Such antioxidants occur at high concentrations in chloroplasts and other cellular compartments, and are crucial for defense of plants against oxidative stress (Noctor and Foyer [1998]; Mittler [2002]). Sato reported that overexpression of *OsAPXa*, an ascorbate peroxidase gene, improves cold tolerance by increasing ascorbate peroxidase activity to reduce the levels of H_2_O_2_ and lipid peroxidation under cold stress (Sato et al. [2011]).

### 2.2 Current criteria for the evaluation of cold tolerance in various rice cultivars

Evaluation of rice cultivars for improved cold tolerance is mainly performed during the seedling and reproductive stages. Cold tolerance at the germination stage is a significant component of rapid seedling establishment and development of a uniform crop stand, especially in direct-seeding production systems (Krishnasamy and Seshu [1989]). Because rice is cultivated in regions that range from tropical to temperate, considerable variation in the extent of cold tolerance is exhibited by seedlings of various *O. sativa* germplasm accessions (Kim and Tai [2011]; Sharifi [2010]). So evaluation of cold tolerance at the seedling stage is important for adaptation to high-altitude or high-latitude locations that can experience cold temperatures at night or early or late in the growing season. Finally, cold tolerance during reproductive stages is critical for pollen survival, seed set, and grain filling to ensure maximal yield (Suh et al. [2010]). Criteria for the evaluation of cold tolerance in rice at different growth stages are summarized in Table 2 and described in detail below.

**Table 2:** Current criteria for evaluation of cold tolerance in rice at different growth stages

**Evaluation criteria**	**Description**	**Temperature and duration**		**Reference**
		**Treatment**	**Recovery**	
**Germination stage**				
Vigor of germination	Vigor of germination (%) = Number of germinated grains/Number of total grains × 100	14°C/7-17 d	-	(Han et al. [2006])
Seedling survival rate	Seedling survival rate (%) = Surviving seedlings/Sprouting seeds × 100, determined when shoots are about 5 mm long.	2°C/3 d	20°C/7 d	(Zhou et al. [2012])
**Seedling stage**				
Fresh weight (FW)	Changes in FW of plants after cold treatment can be used as an indicator of cold damage.	10°C/1-48 h	-	(Bonnecarrère et al. [2011])
Survival rate	Survival rate (%) after cold treatment is calculated as the Number of surviving plants/Total number plants treated × 100	4°C/6 d	26°C/6 d	(Zhang et al. [2011b])
Leaf emergence	New leaf emergence demonstrates maintained vigor and increased growth.	4°C/7 d	25°C/10 d	(Xie et al. [2012])
Seedling growth	1 = dark green seedlings, 3 = light green seedlings, 5 = yellow seedlings, 7 = brown seedlings, 9 = seedlings dead.	9°C/8-14 d	-	(Kim and Tai [2011]; IRRI [2002]; Andaya and Tai [2006])
Leaf growth	A score of 1–3 (tolerant, all leaves normal, no apparent visual injury), or 4–9 (susceptible, all leaves wilted, seedlings apparently dead).	10°C/7 d	25°C/7 d	(Suh et al. [2012])
Metabolic assessments	Metabolites include EL, proline, MDA, and AsA, and GSH.	9°C/1-14 d	-	(Tian et al. [2011]; Zhang et al. [2011a]; Kim and Tai [2011]; Yang et al. [2012])
Enzyme activities	Enzymes include POD, SOD, and CAT, and APX.	4°C/0-4 d	25°C/7 d	(Bonnecarrère et al. [2011]; Huang et al. [2009]; Sato et al. [2001])
**Reproductive stage**				
Spikelet fertility (CGC)	Spikelet fertility is calculated as the ratio of filled grains to the total number of florets, basing on cold greenhouse cultivation.	12°C/6 d	Until maturation	(Sato et al. [2011])
Spikelet fertility (CDWI)	Spikelet fertility is calculated as the same as spikelet fertility (CGC), but the cold treatment is based on cold deep-water irrigation.	18-19°C/~60 d	-	(Shirasawa et al. [2012])

#### 2.2.1 Evaluation of cold tolerance in rice at the germination stage

Germination vigor and seedling survival rate are the two main criteria used for the evaluation of cold tolerance in rice at the germination stage. The vigor of seed germination is recorded at 7 d, 11 d, 14 d, and 17 d following germination at 14°C in the dark.

Germination vigor (%) = (Number of germinated grain/Number of total grain) × 100.

The standard assessment of whether a rice grain has germinated is determined as the point at which the bud length equals half the length of the seed, and the root length equals the seed length (Han et al. [2006]).

The seedling survival rate for cold tolerance is evaluated as follows. When shoots are about 5 mm long, the germinated seedlings are planted in soil and are subjected to cold treatment at 2°C for 3 d, and are then moved to a sunny indoor environment where the temperature is above 20°C to ensure normal growth. Seedling survival rates are assessed after 7 d recovery growth and cold tolerance evaluation indices are calculated as:

Seedling survival rate (%) = surviving seedlings/budding seeds × 100 (Zhou et al. [2012]).

During cultivation, rice seeds are germinated in early spring at the appropriate temperature (usually 32°C), and the germinated seeds are then sown in the field when the shoots are approximately 5 mm long. Thus, rice seedlings are more likely to encounter cold stress after sprouting than during germination. Therefore, seedling survival rate (%) is a more practical criterion for the evaluation of cold tolerance than are traits that are assessed at the germination stage.

#### 2.2.2 Evaluation of cold tolerance in rice at the seedling stage

Both visual and physiological indicators are used to evaluate cold tolerance at the seedling stage in rice. Five criteria are typically used for visual assessment of cold tolerance, including fresh weight, survival rate, new leaf emergence, seedling growth, and leaf growth.

As water loss often occurs concomitantly with plant damage, changes in fresh weight can be used to indicate water loss and growth retardation of rice plants under cold stress (Bonnecarrère et al. [2011]). However, water loss is not always an accurate indicator of cold stress because it can also be affected by traits including plant variety and leaf size, and also by other stressors.

The survival rate (%) (the number of surviving plants divided by the total number of plants treated × 100) is determined after 4°C treatment, which is a severe condition for rice growth that explicitly distinguishes the cold tolerance of cultivars in short time periods ranging from 6 to 7 d (Zhang et al. [2011b]). As this method clearly and efficiently distinguishes degrees of cold tolerance among cultivars and individuals, it is recommended for gauging cold tolerance in the laboratory. Moreover, new leaf emergence can also be used to assess cold tolerance in transgenic rice in terms of maintained vigor and increased growth (Xie et al. [2012]). For some cold-tolerant wild type plants and their transgenic lines, cold stress (4°C treatment) only retards growth rather than causing lethal damage. So, new leaf emergence would be a better choice for distinguishing the cold tolerance of these lines.

The seedling growth scale is derived from the standard evaluation system for rice that was developed by the International Rice Research Institute (IRRI [2002]). After 14 d of cold treatment at 9°C, this assay is performed by scoring seedlings as 1 = dark green seedlings, 3 = light green seedlings, 5 = yellow seedlings, 7 = brown seedlings, 9 = dead seedlings (Kim and Tai [2011]; Andaya and Tai [2006]). Using a similar visual scaling approach, the leaf growth scale is based on the degree of leaf wilting and is scored on a scale of 1–3 (tolerant, all leaves normal, no apparent visual injury) to 4–9 (susceptible, all leaves wilted, seedlings apparently dead) (Suh et al. [2012]). However, unlike the seedling growth scale, in which seedlings are examined immediately after low temperature treatment, the leaf growth assay is based on symptoms that are apparent on the 7th day of the recovery period (Suh et al. [2012]). Because of the moderate treatment (9–10°C), the seedling growth scale and leaf growth assay are often used to show minor differences in cold tolerance between cultivars. Moreover, because the recovery period at 25°C lasts 7 d, the leaf growth assay can better distinguish cultivars with similar cold tolerance than can the seedling growth scale.

As visual ratings are limited by their tendency to be subjective, physiological parameters thus complement the evaluation of cold tolerance in rice. These parameters include measurements of EL, proline, MDA, AsA, and GSH (Tian et al. [2011]; Zhang et al. [2011a]; Yang et al. [2012]), changes in which can be attributed to cold-responsive metabolism. For example, temperate *O. japonica* varieties exhibit less EL, while *O. indica* varieties tend to accumulate higher levels of proline, MDA, AsA, and GSH (Kim and Tai [2011]). The differences in these physiological parameters also indicate the relative degrees of cold tolerance among rice cultivars. Moreover, because MDA is related to the accumulation of ROS, the examination of MDA can indicate whether the cold tolerance in transgenic lines is related to ROS signaling.

In addition, activities of antioxidant enzymes such as peroxidase (POD), superoxide dismutase (SOD), catalase (CAT), and ascorbate peroxidase (APX) can also be used to evaluate cold tolerance in rice. The higher activities of antioxidant enzymes often expressed in transgenic rice lines can indicate their relatively improved cold tolerance compared to wild-type plants (Bonnecarrère et al. [2011]; Huang et al. [2009]). Increased activities of antioxidant enzyme, including OsPOX1, APXa, and related kinase OsTrx23 (Kim et al. [2011]; Sato et al. [2011]; Xie et al. [2012]) may serve to maintain the functions of many other genes during cold stress. Therefore, rather than only physical evaluation of whole-plant cold tolerance, evaluation of antioxidant enzyme activities could both reveal cold-tolerant plants and reveal the mechanisms of cold tolerance.

#### 2.2.3 Evaluation of cold tolerance in rice at the reproductive stage

Exposure to low temperatures during the reproductive stage in rice can cause male sterility and thereby severe yield loss. Cold tolerance at this stage can be evaluated by spikelet fertility based on cold greenhouse cultivation (CGC) or cold deep-water irrigation (CDWI).

To obtain synchronously developing panicles for calculating spikelet fertility using CGC, extra tillers are removed from each greenhouse-grown (26°C/19°C day/night temperature regime) plant at the tillering stage, leaving the main culm. Pollen development is estimated using the auricle distance method (Satake and Hayase [1970]). When the auricle of the flag leaf is approximately 5 cm below the auricle of the penultimate leaf on each plant, the pollen should have undergone meiosis, and each pot is then transferred to a greenhouse maintained at 12°C. After 5–6 d, the cold-treated pots are returned to the warmer greenhouse, where they remain until the plants reach maturity (Andaya and Mackill [2003b]; Suh et al. [2010]). At heading, each plant is tagged with the heading date to verify the developmental stage that pollen had reached after cold treatment, then mean spikelet fertility is calculated for the evaluation of cold tolerance (Sato et al. [2011]).

Spikelet fertility can also be assessed after cold treatment by CDWI. When young panicles begin to differentiate, rice plants are transferred to tanks filled to 20–25 cm depth with water maintained at 18–19°C. Plants are maintained in the cold deep-water irrigation tanks during the entire booting stage. Spikelet fertility is calculated based on the percentage of the number of fertile grains relative to the number of florets (Shirasawa et al. [2012]). Spikelet fertility using CDWI was developed to examine the cold tolerance of rice accessions about 30 years ago, and it is still widely used for selecting cold-tolerant lines because it is highly reliable (Matsunaga [2005]; Shimono et al. [2011]; Shirasawa et al. [2012]).

Both of these methods can be used to select more cold-tolerant rice varieties. Compared to CGC, CDWI exposes plants to a more moderate treatment temperature and a longer treatment period, and is conducted directly in field. Therefore, CDWI is more suitable for distinguishing the cold tolerance of relatively cold-sensitive cultivars and for evaluating the cold tolerance of QTL mapping populations.

### 2.3 QTL identified in various cultivars facilitate the breeding of cold-tolerant rice

Cold tolerance in rice is a quantitative trait controlled by multiple genes. Because it is often difficult to directly associate plant phenotypes with the genes responsible for cold tolerance, marker-assisted selection is an effective means of developing cold-tolerant cultivars (Shirasawa et al. [2012]; Foolad et al. [1999]). The development of molecular markers and linkage maps has made it possible to identify QTL that control cold tolerance in rice (Table 3). QTL analyses have been carried out using rice populations with large levels of genetic variation for cold tolerance (Futsuhara and Toriyama [1969]).

**Table 3:** QTL associated with cold tolerance in rice

**Name of QTL**	**Year**	**Trait for mapping**	**Chromosome no.**	**Reference**
*Ctb1, Ctb2*	2001	Spikelet fertility/Undeveloped spikelet	4	(Saito et al. [2001])
*qCTB2a*, *qCTB3*	2003	Spikelet fertility/Undeveloped spikelet	2, 3	(Andaya and Mackill [2003b])
*qCTS12a*	2003	Seedling growth	12	(Andaya and Mackill [2003a])
*Ctb1*	2004	Spikelet fertility	4	(Saito et al. [2004])
*Dth*, *cl*, *fer*, *pe*, *dc*	2004	Days to heading/Culm length/Spikelet fertility/Panicle neck exsertion/Discoloration	1, 3, 5, 6, 7, 8, 9, 11	(Oh et al. [2004])
*qSV-3-1/2*, −*5*, −*8-1/2*	2005	Seedling growth	3, 5, 8	(Zhang et al. [2005])
*qLVG2*, *qLVG7-2*, *qCIVG7-2*	2006	Vigor of germination	2, 7	(Han et al. [2006])
*qCTS12*	2006	Seedling growth	12	(Andaya and Tai [2006])
*qCTS4*	2007	Seedling growth	4	(Andaya and Tai [2007])
*qCTS-2*	2007	Seedling growth	2	(Lou et al. [2007])
*qLTG3-1*	2008	Vigor of germination	3	(Fujino et al. [2008])
*qCTB-1-1*, −*4-1/2*, −*5-1/2*, −*10-1/2*, −*11-1*	2008	Spikelet fertility	1, 4, 5, 10, 11	(Xu et al. [2008])
*qCTP11*, *qCTP12*	2009	Vigor of germination	11, 12	(Baruah et al. [2009])
*qPSST-3*, −*7*, −*9*	2010	Spikelet fertility/Growth in reproductive stage	3, 7, 8, 9, 11	(Suh et al. [2010])
*Ctb1*	2010	Spikelet fertility/Undeveloped spikelet	4	(Saito et al. [2010])
*qCtss11*	2010	Seedling growth	11	(Koseki et al. [2010])
*qCTB-5-1/2/3*, −*7*	2010	Vigor of germination	5, 7	(Lin et al. [2010])
*qCTS4a*, *qCTS4b*	2012	Seedling growth	4	(Suh et al. [2012])
*qLTB3*	2012	Seed fertility	3	(Shirasawa et al. [2012])

#### 2.3.1 QTL related to cold tolerance at the germination stage

A set of F_2:3_ populations including 200 individuals and lines derived from a cross between the *indica* and *japonica* varieties ‘Milyang 23’ x ‘Jileng 1’ was used to locate QTL for low-temperature vigor of germination (LVG) and to develop a cold-response index for vigor of germination (CIVG). In that study, the QTL *qLVG2* was detected in the region of RM29-RM262 on chromosome 2, and *qLVG7-2* and *qCIVG7-2* were mapped to the region near RM336-RM118 on chromosome 7 (Han et al. [2006]). A major QTL for low temperature germination ability, *qLTG3–1*, was identified by Fujino on chromosome 3 using backcross inbred lines (BIL) derived from a cross between ‘Italica Livorno’ and ‘Hayamasari’ (varieties with vigorous or weak low-temperature germination ability, respectively) (Fujino et al. [2008]). This QTL explains 30% of the total phenotypic variation for low-temperature germination in their mapping population and was thought to be involved in tissue weakening, a key process during seed germination.

Two major QTL (*qCTP11* and *qCTP12*) for cold tolerance at the plumule stage were identified in genetic stocks derived from 34 cultivated (*Oryza sativa*) and 23 wild (*Oryza rufipogon*) rice strains (Baruah et al. [2009]). Both tropical and temperate *japonica* subpopulations, and also annual, intermediate, and perennial *O. rufipogon* types were used for this study (Vaughan et al. [2003]). Cold tolerance was scored based on vigor of germination. In another study, cold tolerance at the bud burst stage (CTB) was evaluated at 5°C in a set of 95 chromosome-segment substitution lines (CSSL), derived from *indica* rice accession 9311 and *japonica* rice cultivar ‘Nipponbare’, which has the genetic background of 9311. In this study, QTL *qCTB-5-1*, *qCTB-5-2*, and *qCTB-5-3* were mapped in the regions of RM267–RM1237, RM2422–RM6054, and RM3321–RM1054, respectively, at positions 21.3 cM, 27.4 cM, and 12.7 cM on rice chromosome 5. Additionally, the QTL *qCTB-7* mapped to a 6.8-cM region near RM11–RM2752 on rice chromosome 7 (Lin et al. [2010]).

#### 2.3.2 QTL related to cold tolerance at the seedling stage

A set of recombinant inbred lines (RIL) that were derived from a cross between M202 and IR50 (*indica*, highly sensitive to cold stress) was used to identify QTL conferring tolerance to cold stress at the seedling stage. Using these RIL, Andaya and Mackill mapped a major QTL, *qCTS12a*, to chromosome 12 that accounted for 41% of the phenotypic variation in seedling growth after cold stress. A number of other QTL with smaller effects have also been detected on eight rice chromosomes (Andaya and Mackill [2003a]). Using RM5746 and RM3103, Andaya and Tai screened 1954 F_5_-F_10_ lines to find recombinants in the *qCTS12* region. Additional microsatellite markers were identified from publicly available genomic sequences and used to fine map *qCTS12* within a region of approximately 87 kb on the BAC clone OSJNBb0071I17. Subsequently, open reading frame analyses delimited the QTL to a region of about 55 kb. The most likely candidates for the gene(s) underlying *qCTS12* are *OsGSTZ1* and *OsGSTZ2* (Andaya and Tai [2006]). *OsGSTZ1* appears to function in improving cold tolerance at the germination stage, as overexpression of this gene in transgenic rice triggered enhanced germination and growth of seedlings at low temperature (Takesawa et al. [2002]). Moreover, the *qCTS4* locus, which is associated with tolerance to yellowing and stunting of rice seedlings, was mapped to chromosome 4 using the same RIL mapping population (Andaya and Tai [2007]).

A set of 282 F_13_ RIL (recombinant inbred lines) derived by single-seed descent from a cross between ‘Lemont’ (*japonica*) and ‘Teqing’ (*indica*) were used to map QTL controlling seedling vigor. A total of 34 QTL for seedling vigor were identified among these RIL. Of the QTL identified, the majority (82%) clustered within five genomic regions, and these were designated *qSV-3-1*, *qSV-3-2*, *qSV-5*, *qSV-8-1*, and *qSV-8-2*, respectively (Zhang et al. [2005]). A major QTL (LOD = 15.09), *qCTS-2*, was detected on chromosome 2 flanked by RM561 and RM341 in doubled haploid (DH) lines that were derived from a cross between a cold-tolerant *japonica* variety (‘AAV002863’) and a cold-sensitive *indica* cultivar (‘Zhenshan97B’) (Lou et al. [2007]). An F_2_ population derived from a cold-tolerant wild relative of rice, W1943 (*Oryza rufipogon*), and a sensitive *indica* cultivar, ‘Guang-lu-ai 4’ (GLA4), were screened by seedling growth to identify QTL that control cold tolerance in rice. A major QTL for cold tolerance at the seedling stage, *qCtss11*, was fine mapped to a 60 kb candidate region defined by markers AK24 and GP0030 on chromosome 11, in which six genes have been annotated. Expression analyses and resequencing of these six candidate genes indicated that the *Os11g0615600* gene is expressed only from the GLA4 allele, and that the *Os11g0615600* gene has a premature stop codon in the GLA4 haplotype. This suggests that either *Os11g0615600* or *Os11g0615900*, or both, might control seedling cold tolerance in this population derived from the cross between W1943 and GLA4 (Koseki et al. [2010]). Moreover, the deduced protein from the *Os11g0615900* gene contains the NB-ARC (nucleotide-binding adaptor shared by APAF-1, R protein, and CED-4) domain, a conserved motif found in disease resistance proteins and involved in hypersensitive response (HR) (DeYoung and Innes [2006]). The further functional studies of the *Os11g0615900* gene under both cold stress and biotic stress would help to define the QTL *qCtss11*, and help discern whether the *Os11g0615900* gene functions in crosstalk between biotic and abiotic stress. Therefore, these genes would be good candidate genes for future comparative functional analyses in cold-sensitive and cold-tolerant populations.

#### 2.3.3 QTL related to cold tolerance at the reproductive stage

Many QTL related to cold tolerance at the reproductive stage have been identified in recent years. Saito and Miura detected two QTL, *Ctb1* and *Ctb2*, on chromosome 4 using a set of near-isogenic lines (NIL) derived from the backcross ‘Kirara397’/’Norin-PL8’/’Kirara397’ (Saito et al. [2001]). The QTL *Ctb1* for cold tolerance as assessed by spikelet fertility was fine mapped to a 56 kb region (Saito et al. [2004]). Moreover, Saito located *Ctb1* within a 17 kb region and finally identified the first gene found to confer cold tolerance at the booting stage of rice. Based on a two-hybrid screen, Saito suggested that the F-box protein encoded by the *Ctb1* gene functions as part of the E3 ubiquitin ligase complex (Saito et al. [2010]). Other QTL related to cold tolerance at the booting stage as assessed by spikelet fertility, *qCTB2a* and *qCTB3*, were mapped using 191 RIL derived from a cross between a temperate *japonica* rice variety, M-202, and a tropical *indica* variety, IR50 (Andaya and Mackill [2003b]). QTL for *Dth* (days to heading), *cl* (culm length), *fer* (spikelet fertility), *pe* (panicle neck exsertion), and *dc* (discoloration) were identified among RIL developed from a cross between the *indica* cultivar, ‘Milyang 23’ and the *japonica* weedy rice, ‘Hapcheonaengmi 3’ (Oh et al. [2004]). A set of NIL with cold tolerance at the booting stage have been developed by backcrossing the strongly cold-tolerant *japonica* landrace, ‘Kunmingxiaobaigu’ (KMXBG) to the cold-sensitive *japonica* cultivar, ‘Towada’, as the pollen recipient parent. Eight QTL based on variation in spikelet fertility, *qCTB-1-1*, *qCTB-4-1*, *qCTB-4-2*, *qCTB-5-1*, *qCTB-5-2*, *qCTB-10-1*, *qCTB-10-2* and *qCTB-11-1*, were mapped in this population on chromosomes 1, 4, 5, 10, and 11, respectively. All of the alleles for cold tolerance were contributed by KMXBG, and the marker intervals containing these QTL were narrowed to 0.6–5.6 cM (Xu et al. [2008]). QTL analysis with simple sequence repeat (SSR) markers and composite interval mapping has identified three main-effect QTL assessed by spikelet fertility and growth during the reproductive stage, including *qPSST-3*, *qPSST-7*, and *qPSST-9*, respectively, on chromosomes 3, 7, and 9. In addition, a new source of cold-tolerance measured by spikelet fertility, line IR66160-121-4-4-2, was used as the pollen donor parent in a cross with a cold-sensitive cultivar, ‘Geumobyeo’, to produce 153 F_8_ RIL for QTL analysis (Suh et al. [2010]). Another QTL for cold tolerance, *qLTB3*, was identified on the long arm of chromosome 3 from the cold-tolerant breeding line ‘Ukei 840’ in F_2_ and BC_1_F_2_ populations from crosses between ‘Ukei 840’ and ‘Hitomebore’. The cold tolerance of ‘Ukei 840’ is derived from the Chinese cultivar ‘Lijiangxintuanheigu’ (Shirasawa et al. [2012]). Cold tolerance related to *qLTB3* in these lines was assessed by seed fertility.

Taken together, QTL for cold tolerance have been detected in rice at the seedling stage (Andaya and Mackill [2003a]; Zhang et al. [2005]), booting stage (Xu et al. [2008]), bud burst stage (Lin et al. [2010]), and plumule stage (Baruah et al. [2009]), suggesting that cold tolerance could be both developmentally regulated and growth-stage specific (Suh et al. [2012]; Foolad and Lin [2001]).

## 3 Conclusions

Systematic studies have been carried out to improve our understanding of the physiological and genetic basis of cold tolerance in rice, which will promote the development of rice cultivars with improved cold tolerance. Cold stress interferes with metabolism and initiates changes in various physiological properties of plants. In rice, these changes are part of the cold-sensing system that initiates the cold-responsive signaling network. Reduced chlorophyll content and fluorescence (Fv/Fm) triggered by cold stress indicate injury to photosynthesis, as increased ROS and MDA subsequently mediate cold damage and cold sensing. Soluble sugars, proline, and antioxidants then accumulate and protect rice plants from further damage. Moreover, measurements of these physiological properties can also be used to evaluate the extent of cold tolerance exhibited by various rice cultivars to identify genes controlling cold tolerance for breeding purposes.

Currently, there are many criteria for the evaluation of cold tolerance; but is there a “gold standard” among techniques for assessing cold tolerance of a specific rice cultivar? The appropriate technique may depend on a number of factors, so a strong correlation between the measured trait and the ultimately desired trait, whether survival or yield after cold stress, is very important. Although a decrease in fresh weight can indicate cold stress, water loss is not always an accurate indicator of cold stress, because plant variety, leaf area, and other stressors can also affect water content measurements. Visual evaluations of cold stress using the seedling growth scale are performed immediately after a 14 d cold treatment at 9°C, while those using leaf growth are performed after 7 d of recovery from 9°C cold stress. These techniques, particularly the latter, can help make fine distinctions in cold tolerance between varieties or individuals. Seedling survival rate (%) can be a more practical criterion for cold tolerance than germination-stage cold tolerance because seedlings are more likely to actually experience cold stress in the field. Seedling survival would also be useful for addressing the discrepancies between greenhouse- and field-based results. For sublethal cold stress, such as 4°C cold stress that merely slows growth but does not cause severe damage, new leaf emergence is a better choice for distinguishing the cold tolerance of lines stressed at 4°C. But an important disadvantage of all germination- and seedling-stage assays is that they do not necessarily predict reproductive-stage cold tolerance, or may do so in a variety-specific manner.

For selection of appropriate parents for cold tolerance breeding, evaluation of cold tolerance at the reproductive stage is important. And because it results in strong selection, the examination of spikelet fertility after CGC seems to be an ideal approach for improving cold tolerance relatively quickly. To identify cold-tolerance related QTL that operate at the reproductive stage, the examination of spikelet fertility after CDWI is particularly useful because it is conducted directly in the field. Mechanistic studies to evaluate metabolic parameters and enzyme activities can be valuable for researchers who are interested in the functional characterization of genes related to cold response, because changes in metabolism and enzyme activities are useful for revealing the role of genes that function in the cold-sensing and cold-response networks.

The development of molecular markers and linkage maps has allowed detection of many QTL related to cold tolerance at various growth stages. Most QTL have been identified in mapping populations derived from crosses between varieties or accessions derived from *O. japonica* and *O. indica*, and between cultivated rice and its wild relatives. The future application of these QTL to MAS will significantly accelerate the breeding of cold-tolerant rice for temperate environments and high-altitude areas. However, the gap between greenhouse research and field application is a major concern in cold tolerance research. To resolve this problem, more researchers are evaluating cold tolerance using criteria that are conduct directly in the field, such as spikelet fertility after CDWI, to identify cold-tolerance related QTL. Moreover, Suh and Jena used both greenhouse and direct field methods to cross-validate their results (Suh et al. [2010]; Jena et al. [2012]), a positive trend for cold tolerance research in rice. Furthermore, in order to characterize and confirm the function of QTL identified in the greenhouse, Saito and Koseki conduct expression analysis or transgenic studies of candidate genes (Saito et al. [2010]; Koseki et al. [2010]). These approaches help to validate the function of these QTL in cold-tolerance and are necessary before initiating selection. In addition to these QTL and molecular markers, the mapping populations that have been established and used in mapping projects are valuable tools that are directly useful in cold tolerance breeding. In the future, the integration physiological mechanistic studies of cold tolerance and QTL identification will accelerate the improvement of rice for the traits related to cold tolerance.

## Abbreviations

QTL: Quantitative trait loci

ROS: Reactive oxygen species

MDA: Malondialdehyde

MAS: Molecular marker-assisted selection

EL: Electrolyte leakage

GC-MS: Gas chromatography–mass spectrometry

ABA: Abscisic acid

ABRE: ABA-responsive elements

ABF: ABRE-binding bZIP transcription factor

AsA: Ascorbic acid

GSH: Glutathione

POD: Peroxidase

SOD: Superoxide dismutase

CAT: Catalase

APX: Ascorbate peroxidase

CGC: Cold greenhouse cultivation

CDWI: Cold deep-water irrigation

## Competing interests

The authors declare that they have no competing interests.

## Authors’ contributions

QZ, QC, and SW performed the analysis, and prepared the figure and tables. QZ, YH, and ZW designed and wrote the paper. All authors read and approved the final manuscript.
